# Comparison of response rates on invitation mode of a web-based survey on influenza vaccine adverse events among healthcare workers: a pilot study

**DOI:** 10.1186/s12874-018-0524-8

**Published:** 2018-06-20

**Authors:** Xiaochen Tai, Alanna M. Smith, Allison J. McGeer, Eve Dubé, Dorothy Linn Holness, Kevin Katz, Linda McGillis Hall, Shelly A. McNeil, Jeff Powis, Brenda L. Coleman

**Affiliations:** 1 Sinai Health System, Mount Sinai Hospital, Toronto, ON Canada; 2 University of Toronto, Toronto, ON Canada; 3 National Public Health Institute of Quebec, Quebec City, QC Canada; 4 Laval University, Quebec City, QC Canada; 5 St. Michael’s Hospital, Toronto, ON Canada; 6 North York General Hospital, Toronto, ON Canada; 7 Dalhousie University, Halifax, NS Canada; 8 Nova Scotia Health Authority, Halifax, NS Canada; 9 IWK Health Centre, Halifax, NS Canada; 10 Canadian Center for Vaccinology, Halifax, NS Canada; 11 Michael Garron Hospital, Toronto, ON Canada

**Keywords:** Web-based survey, Response rate, Invitation mode, Email invitation, Postal invitation, Influenza

## Abstract

**Background:**

Web-based surveys have become increasingly popular but response rates are low and may be prone to selection bias. How people are invited to participate may impact response rates and needs further study as previous evidence is contradictory. The purpose of this study was to determine whether response to a web-based survey of healthcare workers would be higher with a posted or an emailed invitation. We also report results of the pilot study, which aims to estimate the percentage of adults vaccinated against influenza who report recurrent systemic adverse events (the same systemic adverse event occurring successively following receipt of influenza vaccines).

**Methods:**

The pilot study was conducted in November 2016 in Toronto, Canada. Members of a registry of adults (18 years and older and predominantly healthcare workers) who volunteered to receive information regarding future studies about influenza were randomly assigned to receive either an email or postal invitation to complete a web-based survey regarding influenza vaccinations. Non-respondents received one reminder using the same mode of contact as their original invitation.

**Results:**

The overall response rate was higher for those sent the invitation by email (34.8%) than by post (25.8%; *p* < 0.001) and for older versus younger participants (*p*_trend_ < 0.001). Of those who responded, 387/401 had been vaccinated against influenza at least once since adulthood. Of those responding to the question, 70/386 (18.1%) reported a systemic adverse event after their most recent influenza vaccine including 22 (5.7%) who reported a recurring systemic event. Systemic adverse events were reported more often by males 18–49 years old than by other groups (*p* = 0.01). Recurrent systemic adverse events were similar by age and sex with muscle ache being the most commonly reported recurrent reaction. More respondents who reported only a local adverse event (93.1%) planned to be vaccinated again next year than those with a systemic adverse event (69.7%; *p* = 0.04).

**Conclusions:**

In this convenience sample of registry volunteers, response rates were generally low, but were higher for the emailed than posted invitations and for older than younger adults.

## Background

Web-based surveys are becoming increasingly popular with the expansion of access to the internet [1]. Advantages over paper-based surveys include reduced cost [2, 3], user convenience for automatic skip patterns [4], and reduced transcription error and data entry costs as responses are entered directly into a database [5]. However, technical issues can frustrate potential participants if sites are not easily accessible, properly designed, and well maintained. Emailed invitations to web-based surveys have many attractive advantages including direct links to survey sites, ease of sending and responding, and lower costs [6]. However, delivery and open rates suffer due to incorrectly recorded or outdated addresses, blockage by spam filters, and lack of perceived legitimacy [7]. Also, since email addresses may not be available for all members of the population of interest, there are concerns about selection bias [8]. In contrast, postal invitations may appear more legitimate and attract greater attention, but are somewhat more burdensome, requiring more participant effort when asked to respond to an online questionnaire [9], Postal invitations can also be more costly to the researchers, can suffer from non-delivery due to outdated or incorrect addresses and low response due to perceived importance.

Previous evidence comparing the impact of different invitation modes on response rates of web-based and paper-based surveys is contradictory. Several research reports have demonstrated the higher response rates to posted invitations for paper-based surveys compared to emailed invitations for web-based surveys [1, 10], with the lower rates seen for postal invitations to web-based surveys [11]. Conversely, two studies of student populations reported higher rates of response to emailed invitations for web-based surveys than posted invitations for paper-based ones [12–14] suggesting possible age and cohort effects.

In a similar contradictory manner, posted invitations to web-based surveys had higher [6, 8] or similar [6, 9, 15, 16] response rates to emailed invitations for those same surveys. See Dykema et al. [6] for a more extensive review. Meanwhile, a large campus-wide project reported different results across subpopulations: emailed invitations yielded a significantly higher response rate to the web-based survey than postcard invitations for faculty (32.6% vs. 20.9%; *p* < 0.01), while there was no difference in response rates by invitation mode for non-faculty staff or for students after two invitations [17]. Dykema et al. [6] reported that response to mailed invitations to an online survey was more representative of the invitation group than those invited by email.

Influenza vaccination is strongly recommended in people working in healthcare facilities and an increasing number of facilities require employees to be vaccinated as a condition of employment or to be vaccinated or wear a surgical mask while providing care to patients during the influenza season. Despite evidence supporting vaccination against influenza for healthcare workers to protect their vulnerable patients [18, 19], only about 50% are vaccinated annually in Canada [20, 21]. Meanwhile, vaccine hesitancy and refusal appears to be increasing [22], with adverse events being a top concern of vaccine hesitant people. Numerous studies confirm higher rates of systemic adverse events in people receiving influenza vaccine than in those receiving a placebo [23], but there are no studies on the recurrence of systemic adverse events nor the effect that recurring systemic adverse events have on healthcare workers’ decisions to be vaccinated again. Since annual revaccination is necessary due to the drifting and shifting of the circulating strains of influenza, these vaccines are distinct from other vaccination programs making the adverse events of particular importance.

The primary objective of this study was to determine the impact of invitation mode (emailed vs. posted) on response rates for a web-based survey regarding influenza vaccination. A secondary objective was to estimate the percentage of people receiving the influenza vaccine who reported recurrent systemic adverse events.

## Methods

### Study participants

The sample for this study was drawn from members of a registry maintained by the investigator. The registry consists of adults aged 18 years and older, mainly (84.4%) healthcare workers, who participated in previous studies conducted by the Infectious Disease Epidemiology Research Unit at Mount Sinai Hospital, Toronto, Canada. The registry contains the volunteer’s name, year of birth, sex, and email and postal addresses. Eligibility for this study was limited to registrants with both an email and a postal address. The study was approved by the Sinai Health System research ethics board (REB #16–0267-E).

### Study design and methodology

Eligible individuals were randomly assigned to one of eight groups. Four groups received an invitation by email with a direct link to the survey site while the other four groups received an invitation by post with the URL to the survey site included in the letter. Each invitation arm was randomized to receive one of four versions of the questionnaire: short or long with either open- or close-ended questions. A unique 6-digit personal identification (PID) code was assigned to each individual using a random number generator and used to ensure that only invited people were able to participate and to track responses so responding individuals did not receive unnecessary reminders. Individuals accessing the survey site first landed on the consent page. If they consented, they were brought to the PID code verification page. Once the code was verified, they were taken directly to the appropriate questionnaire. All questionnaires were implemented using SimpleSurvey® (OutSideSoft Solutions Inc., Quebec).

Invitations asked potential participants to help us learn “how to best ask questions” and their experiences with adverse events following immunization with influenza vaccines. The visual design and subject matter was consistent across the four versions of the questionnaire. Questionnaire version distinctions were made on the basis of i) overall length: short (24 questions) versus long (additional 4 questions) and ii) response format: open-ended versus close-ended (to five (short version) or six (long version) questions). Questions were developed through adaptation (with permission) of existing questionnaires including Nichol and Hauge (1997), Feemster et al. (2011), and Opel et al. (2011) [24–26]. Questions included age, sex, occupation, work site, as well as influenza vaccination history, factors affecting the decision to be vaccinated, experience with side effects, and impact of side effects. To determine if there were signals that might predict adverse events in certain populations, there was a series of questions asking about non-influenza vaccines received and resulting side effects, as well as allergies or sensitivities to medications, foods, or environmental allergens. Skip logic was used to present respondents with follow-up questions based on previous responses. Open-ended responses were categorized by one person (XT) using the closed-ended items as a guide.

### Data collection

The initial invitations were sent by post or email on November 22, 2016 to allow for the maximum number of respondents to be vaccinated against influenza for the season but to allow for reminders and responses before the Christmas holiday vacation period. Each invitation was personalized (e.g., “Dear Jane”) and contained a direct URL to the survey, a brief introduction to the study to increase salience, and the PID code. The invitation emails were sent using the SimpleSurvey® interface which generated a list of email addresses to which the invitations had failed to send (e.g., address errors, inactive accounts). If errors were identified and a second email address was listed in the registry, a second attempt was made via email; if not, no further contact was attempted. If an invitation letter was returned by the postal service (e.g., invalid address), no further attempt was made to contact the subject. The research assistant answered telephone and email queries from potential respondents who had questions about the study or problems accessing the survey site.

Reminder emails were sent to all non-respondents seven days after the initial email while reminder letters were sent 14 days after the initial letter. The reminder letter was delayed by 7 days compared with the reminder email to allow for delivery by the postal system, which is commonly 2–4 business days. The online surveys were deactivated on December 28, 2016 and response data were downloaded the same day.

### Statistical analysis

Response rates by invitation mode (i.e., post vs. email) were calculated based on the final disposition codes defined by the American Association for Public Opinion Research (AAPOR) using response rate 2 (RR2), which counts both complete and partial responses in the numerator [27].

$$ RR2=\frac{\left(I+P\right)}{\left(I+P\right)+\left(R+ NC+O\right)+\left( UH+ UO\right)} $$

(Abbreviation: RR, response rate; I, complete interview; P, partial interview; R, refusal and break-off; NC, non-contact; O, other; UH, unknown if household/occupied HU; UO, unknown, other.)

Adverse events following influenza vaccination were categorized into four groups: no adverse event, local adverse event only, systemic adverse event (most recent only), and recurrent systemic adverse event. An adverse event was defined as any untoward, unfavourable, or unintended symptom or disease following influenza immunization. A local adverse event was defined as any local reaction at the injection site including pain, soreness, numbness, bruising, redness, or swelling. A systemic adverse event was any of: upper respiratory illness (with or without sore throat, coughing, stuffed or runny nose, etc.), fever/feverishness, headache, new joint pain, malaise/feeling generally unwell, new & unexplained muscle aches, tiredness/fatigue or sweating within 7 days, a rash with onset within 48 h, or an allergic reaction with onset within 24 h of vaccine receipt [28]. A recurrent systemic adverse event was defined as at least one of the same systemic adverse event symptoms occurring following the receipt of the two most recent influenza vaccines. Respondent’s ages were categorized into four groups (18–34; 35–49; 50–64; 65+ years).

Chi-square or Fisher’s exact tests were used as appropriate while non-parametric trend tests were used to assess by ordered age groups. All tests were two-tailed with a *p*-value of < 0.05 considered statistically significant using Stata SE version 11 (StataCorp, 2009). Multiple logistic regression analyses were used to determine the association between invitation mode and response rate. Regression diagnostics were performed to identify influential observations and outliers using Pearson residuals, deviance residuals and Pregibon leverage graphs. Since effect modification by sex was detected, sex-specific regression analyses were carried out adjusted for age.

## Results

### Study sample

**Fig. 1:**
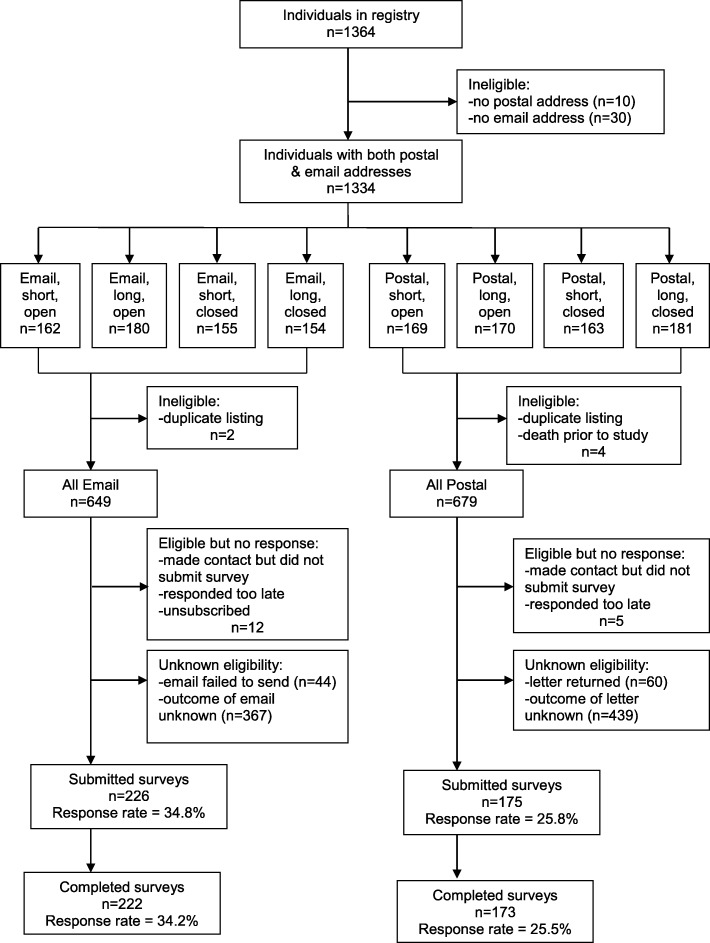
Survey completion flow chart

As shown in Fig. 1, of 1364 people in the registry, 1334 participants aged 22–94 years had both an email and postal address and were randomized to 8 groups by invitation mode, survey length (long vs. short), and response type (open- vs. close-ended). There was no significant difference by age or sex in those who were and were not eligible to be randomized. After excluding 6 ineligible individuals, there were 649 and 679 participants eligible for contact by email and post, respectively. The eligible sample had a median age of 48 years (95% CI: 47, 49) and 80% were female, which is reflective of the healthcare personnel in Canada [29].

There were no differences by median age (48 vs. 48 years; *p* = 0.89) or sex (78.7% vs. 81.6% female; *p* = 0.18) between the two invitation mode groups (email and post, respectively).

There were 559 (41.9%) potential participants with two email addresses available in the registry. After invitations were sent, 67 (10.3%) initial emails and 60 (8.8%) letters were returned as improperly addressed (*p* = 0.35). Twenty-three (76.7%) emailed invitations were successfully re-sent to an alternate email address. There was ultimately no statistically significant difference in the percentage of returned invitations by mode (44 (6.8%) email vs. 60 (8.8%) post) (*p* = 0.17).

### Response rates

As shown in Table 1, the overall response rate was higher for those sent the invitation by email (34.8%) than by post (25.8%; *p* < 0.001). Older participants were more likely to respond to the survey than younger ones, overall (median age 53 vs. 46 years, *p* < 0.001) and by invitation mode (*p*_trend_ < 0.001). Participants responding to emailed invitations were younger (median of age: 51 years) than those who responded to postal invitations (55 years; *p* = 0.005). Although males and females were equally likely to respond overall, females were more likely to respond to the email (36.0%) than the posted letter (24.4%; *p* < 0.001). This was driven by females younger than 50 years of age who were significantly more likely to respond to the email versus the postal invitation (32.1% vs. 15.9%, *p* < 0.001) while there was no difference for older women. Males 50 years and older were more likely to respond to the posted invitation (51.7%) than females in that age group (34.8%; *p* = 0.02) while there was no difference in response rates to emailed invitations (41.1 and 35.6%, respectively).

**Table 1:** Comparison of response rates by invitation mode, age group and sex

	Total		Email invitation		Postal invitation		*p-value*
	Number sent	Response rate^a^	Number sent	Response rate^a^	Number sent	Response rate^a^	Email vs post
Overall	1328	30.2	649	34.8	679	25.8	*< 0.001*
Age group^b^							
18–34 yrs	202	17.8	101	23.8	101	11.9	*0.03*
35–49 yrs	504	25.4	243	34.2	261	17.2	*< 0.001*
50–64 yrs	475	36.4	230	40.0	245	33.1	*0.12*
≥ 65 yrs	131	48.1	65	40.0	66	56.1	*0.07*
*p-value (trend)*		*< 0.001*		*0.01*		*< 0.001*	
Sex^c^							
Female	1063	29.9	509	36.0	554	24.4	*< 0.001*
Male	263	31.6	138	31.2	125	32.0	*0.88*
Age and sex^b,c^							
Female 18–49 yrs	564	23.6	268	32.1	296	15.9	*< 0.001*
Male 18–49 yrs	140	22.1	74	28.4	66	15.2	*0.06*
Female ≥50 yrs	489	37.8	236	41.1	253	34.8	*0.15*
Male ≥50 yrs	117	43.6	59	35.6	58	51.7	*0.08*

vs: versus; yrs.: years

^a^AAPOR response rate2 (number completed + number partial completes/sample size)

^b^16 respondents had no data for age

^c^2 respondents had no data for sex

As shown in Table 2, there were some statistically significant differences in response rate by the length and/or format (open-ended vs. close-ended) of the questionnaire, with those randomized to the longer and open-ended questionnaire more likely to respond if they were invited by email. However, since the potential participants were not aware of the questionnaire they would receive, the differences are likely due to statistical chance. There was no statistical association between the length and type of questionnaire and the likelihood of providing an incomplete survey. Of the 6 (1.5% of 401) respondents who submitted partially completed surveys, 2 received long, open-ended, 3 received long close-ended, and 1 received short open-ended versions (*p* = 0.53).

**Table 2:** Comparison of response rates by invitation mode and survey type

	Total		Email invitation		Postal invitation		*p-value*
	Number sent	Response rate^a^	Number sent	Response rate^a^	Number sent	Response rate^a^	Email vs postal
Overall	1328	30.2	649	34.8	679	25.8	*< 0.001*
Length & type							
Short, closed	316	27.9	154	28.6	162	27.2	*0.78*
Short, open	330	31.2	162	36.4	168	26.2	*0.05*
Long, closed	334	31.4	154	35.1	180	28.3	*0.19*
Long, open	348	30.2	179	38.6	169	21.3	*< 0.001*

^a^AAPOR response rate2 (number completed + number partial completes/sample size)

For women, the odds of responding to the survey was higher (OR 1.77) by email than post (*p* < 0.001). On the other hand, there was no difference in the odds of responding by email or post for males while holding the effects of age constant (*p* = 0.90). While holding the method of response (email verus post) constant, both older men and older women were more likely to respond than younger ones (see Table 3).

**Table 3:** Association between invitation mode and response rate, stratified by sex

	Odds Ratio	95% Confidence Interval	*p*-value
Male			
Invitation mode			
Post	Referent		
Email	0.97	(0.56, 1.68)	0.90
Age group^a^			
18–34 years	Referent		
35–49 years	6.87	(1.55, 30.4)	0.011
50–64 years	10.43	(2.33, 46.7)	0.002
≥ 65 years	21.20	(4.50, 99.8)	0.000
Female			
Invitation mode			
Post	Referent		
Email	1.77	(1.35, 2.32)	< 0.001
Age group^a^			
18–34 years	Referent		
35–49 years	1.29	(0.83, 2.01)	0.26
50–64 years	2.23	(1.44, 3.43)	< 0.001
≥ 65 years	3.19	(1.81, 5.64)	< 0.001

^a^16 respondents had no data for age

**Fig. 2:**
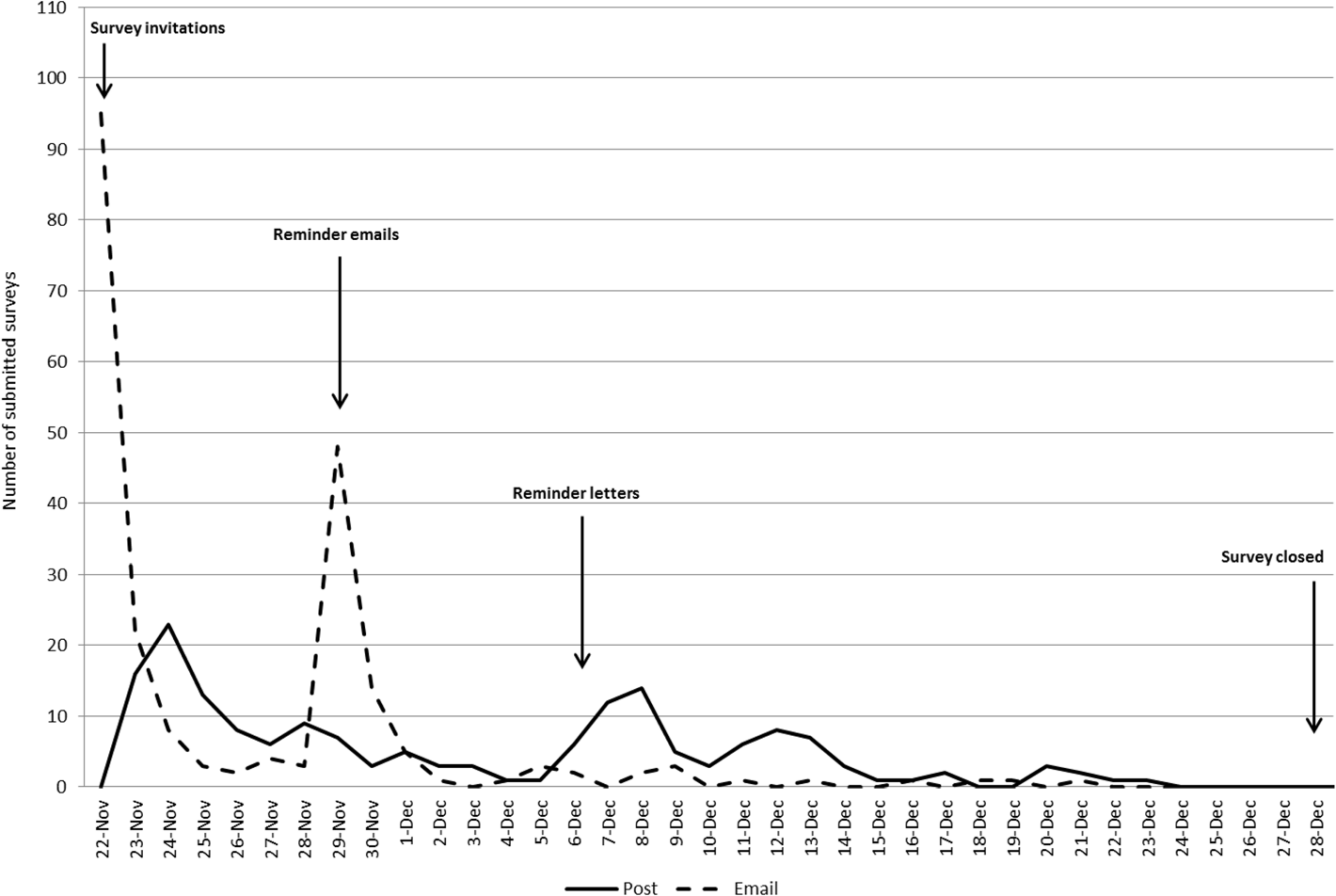
Survey response time by mode of invitation

As shown in Fig. 2, the number of responses from the emailed invitation reached a peak on the day the first invitation was sent with a second peak on the day the reminder emails were sent. In comparison, the peak for the posted invitations was 2 days after the first letters were sent with a second peak 2 days after the reminder letters were sent. The response rate after the initial invitation (up to date of second invitation) was 21.3 and 14.4% for those who received email and postal invitations, respectively (*p* < 0.001). Older participants were more likely to respond after initial invitations than younger groups (*p* < 0.001) but there were no difference by sex (*p* = 0.27).

### Adverse events following immunization

Of the 401 people who completed a questionnaire, 96.5% had been vaccinated against influenza at least once during adulthood and 76.0% were vaccinated every year since their initial vaccination. Of those who had not been vaccinated during adulthood, the main reasons stated were: the effectiveness of the vaccines (*N* = 5), inconvenience (*N* = 3), and not feeling at high risk of getting influenza (*N* = 3). The top reasons for not being vaccinated every season were: the limited effectiveness of the vaccines (*N* = 28), inconvenience (*N* = 22), concern about side effects from influenza vaccines (*N* = 21), and not feeling at high risk of getting influenza (*N* = 14).

Of the 387 previously vaccinated participants, 100 (25.8%) reported an adverse event following receipt of their most recent influenza vaccine including 70 (18.1%) who reported systemic event(s) including muscle ache (*N* = 27), acute upper respiratory illness (*N* = 26), headache (*N* = 25), fatigue (*N* = 21) and malaise (*N* = 17). Recurrent systemic adverse events (same systemic event after their two most recent vaccinations) was reported by 22 of 387 participants (5.7%) and included muscle aches (*N* = 13), fatigue (*N* = 9), headache (*N* = 8), malaise (*N* = 7) and acute upper respiratory illness (*N* = 7).

As shown in Table 4, there were no differences (at *P* < 0.025, to adjust for multiple testing) in the percentage of respondents reporting recurrent systemic adverse events by invitation mode, question format (open vs. closed), length of the survey, age, sex, or adverse event recall of non-influenza vaccines. Of the participants who recalled their experience with non-influenza vaccines, 67.8% (208/307) reported no adverse events after any vaccines while 8.1% (25/307) reported an adverse events (local or systemic) after both influenza and non-influenza vaccinations. There was also no correlation between known allergies or sensitivities and the occurrence of recurrent systemic adverse events.

**Table 4:** Adverse events following influenza vaccination by age group, sex and reaction to non-influenza vaccines

		Adverse event after influenza vaccine				
	Total	No AE	Local AE only	Systemic AE	Recurrent systemic AE	*p-value*
Overall	386 (100.0)^a^	287 (74.4)	29 (7.5)	48 (12.4)	22 (5.7)	
Invitation Mode						
Email	218 (56.5)	153 (70.2)	15 (6.9)	34 (15.6)	16 (7.3)	
Post	168 (43.5)	134 (79.8)	14 (8.3)	14 (8.3)	6 (3.6)	*0.05*
Length						
Short version	180 (46.6)	121 (67.2)	18 (10.0)	31 (17.2)	10 (5.6)	
Longer version	206 (53.4)	166 (80.6)	11 (5.3)	17 (8.3)	12 (5.8)	*0.01*
Format						
Closed-ended	187 (48.4)	141 (75.1)	15 (8.0)	18 (9.6)	13 (7.0)	
Open-ended	199 (51.6)	146 (73.4)	14 (7.0)	30 (15.1)	9 (4.5)	*0.32*
Age^b^						
18–49 yrs	155 (40.3)	106 (68.4)	11 (7.1)	28 (18.1)	10 (6.5)	
≥ 50 yrs	230 (59.7)	180 (78.3)	18 (7.8)	20 (8.7)	12 (5.2)	*0.05*
Sex						
Female	303 (78.5)	224 (74.0)	23 (7.6)	35 (11.6)	21 (6.9)	
Male	83 (21.5)	63 (75.9)	6 (7.2)	13 (15.7)	1 (1.2)	*0.17*
Age^b^ and Sex						
Female 18–49 yrs	123 (32.0)	85 (69.1)	9 (7.3)	19 (15.5)	10 (8.1)	
Male 18–49 yrs	32 (8.3)	21 (65.6)	2 (6.3)	9 (28.1)	0	
Female ≥50 yrs	180 (46.8)	139 (77.2)	14 (7.8)	16 (8.9)	11 (6.1)	
Male ≥50 yrs	50 (13.0)	41 (82.0)	4 (8.0)	4 (8.0)	1 (2.0)	*0.11*
AE after non-influenza vaccines						
Yes	58 (15.0)	33 (56.9)	11 (19.0)	7 (12.1)	7 (12.1)	
No	248 (64.3)	208 (83.9)	10 (4.0)	21 (8.5)	9 (3.6)	
Don’t recall/No answer	80 (20.7)	46 (57.5)	8 (10.0)	20 (25.0)	6 (7.5)	*< 0.001*

Abbreviations: AE: Adverse event; yrs.: Years

*P < 0.025 when comparing recurring systemic adverse events by factor

^a^respondent had no description of his/her adverse event

^b^respondent provided no data for age

A higher percentage of respondents who reported only a local adverse event (27/29 or 93.1%) than those who reported a systemic adverse event (46/66 or 69.7%; *p* = 0.04) planned to be vaccinated again next season. Of the 66 people who reported systemic adverse events, an equal percentage of those with an event after only their most recent vaccination (29/45 or 64.4%) and those with recurring events (17/21 or 81.0%; *p* = 0.38) planned to be vaccinated again next season. Respondents whose adverse event affected their daily activities or required a medical consultation were significantly less likely to plan to be vaccinated next year (12/25 or 48.0%) than those with adverse events that did not (61/70 or 87.1%; *p* < 0.001). Participants who reported no side effects were not asked about their intentions to receive the influenza vaccination next season; a change will be made to the final survey to correct this oversight.

## Discussion

The overall response rate to our web-based survey was low (30%), but higher for subjects 50 years and older compared with younger adults. Response rates were also higher for those sent an email invitation and email reminder with a clickable link to the web-based survey than for those sent a postal invitation and reminder which contained the survey site address which then had to be entered into the internet browser’s address bar.

Our response rate was consistent with responses to other surveys conducted with health professionals [1, 6, 11]. McPeake, Bateson and O’Neil hypothesized that health professionals may be overburdened by surveys and may complete only surveys they feel are absolutely necessary or are of particular interest to them [30]. The level of interest or salience may explain why subjects 50 years and older were more likely to respond to the survey than younger ones. In Canada, people 50 years of age and older are more likely to be vaccinated against influenza than younger ones [21]. Although one web-based survey about knowledge of arts education reported that participants 50 years and older were significantly more likely than their younger counterparts to respond [31], many others reported no difference in response rates by age [10, 12, 14]. However, the registry was small (only 1328 eligible) and results should be interpreted with caution.

Contrary to the current evidence that emailed invitations produce a lower or non-superior response rate to posted invitations [6, 8, 15, 16], findings from our study suggest that emails may yield higher response rates in some populations. This finding may reflect our registry population who, for the most part, were healthcare workers who had participated in studies requiring the use of the internet. Unfortunately, with the increased use of email for advertising, phishing, and malware, it is possible that response rates will decrease as has been the experience with telephone surveys [32]. It is likely that our response rates would have been better if we had used more reminders, particularly if those reminders were done by post [33]. Newberry and Israel reported that response rates for adults in a state-run client registry jumped from < 30% after the third contact to over 55% after the fifth [34]. Although the first three modes of contact varied (post only, post followed by 2 emails, or three emails), the final two contacts in each arm were mailed letters with questionnaires.

Non-delivery rates for both contact modes were an issue in this study; one that we were unable to accurately measure. Although there was no difference in the percentage of known non-delivery by mode, we have no knowledge of how many invitations were filtered prior to delivery due to spam-filters [35] or were not returned by the current resident at the mailing address. We were also unable to ascertain how many individuals did not respond due to disinterest [15] or lack of engagement and/or salience.

Not unexpectedly, the rate of recurrent systemic adverse events following influenza immunization (5.7%) was higher than reported by passive surveillance systems. Vellozzi, Burwen, et al. reported that of the 18,245 events following influenza vaccination reported to the Vaccine Adverse Event Reporting System in the USA, 109 (0.6%) were recurrent with 16 (0.09%) of them being recurrent and systemic [36]. We found no association between recurrent systemic adverse events and respondent age, sex or reactions to non-influenza vaccines or allergens. However, the number of respondents was low and this result should be interpreted with caution. The most common recurrent adverse events were muscle aches and fatigue. This finding corresponds with results from an influenza vaccine safety surveillance study in which Canadian healthcare workers reported fatigue/myalgia as the most common systemic symptom interfering with daily activities or requiring healthcare consultation [37].

The fear of adverse events is an often-stated reason for rejecting the annual influenza vaccine [38, 39]. In our study, respondents who experienced a systemic adverse event were significantly less likely to plan to be vaccinated against influenza in the future than those who had only a local reaction. This is similar to Turkish medical residents of whom 20/29 (69%) experiencing side effects were not planning to be vaccinated the following season compared with 13% of those who did not [40]. Contrary to this, a 1980s study found no difference in the percentage of outpatients who were actually revaccinated the following year based on whether they had a local or systemic reactions or neither [41]. Of note, participants who experienced recurring systemic adverse events were equally likely to plan future influenza vaccinations as people who experienced a systemic adverse event in the most recent season only. This suggests that people may overcome their initial decision regarding revaccination once the chance of an adverse event is weighed against not being protected against influenza the following season.

Strengths of this study include random allocation of registrants and equivalent numbers and types of contacts by invitation mode. One limitation is that the registry was seldom updated which increased the probability for non-delivery. Although this affected the overall response rate, it would not have had an impact on the response by invitation mode. The registry was limited to volunteers in previous influenza studies, which may have increased the overall response rate due to interest in the topic. The high rate of previous influenza vaccination is a product of the volunteers within the registry. We were unable to assess response rates by factors beyond age and sex as the registry had no information on occupation or other factors.

## Conclusions

In our population, emailed invitations yielded a higher response rate and shorter response times than posted invitations. Older respondents were more likely to respond to the survey in general than their younger counterparts while participants responding to emailed invitations were younger. The most common recurrent systemic adverse events were myalgia and fatigue.

## Abbreviations

AAPOR: American Association for Public Opinion Research
I: Complete interview
NC: Non-contact
O: Other
P: partial interview
PID: Personal Identification (code)
R: Refusal and break-off
RR2: AAPOR’s standard definition for Response Rate 2
UH: Unknown if household/occupied HU
UO: Unknown, other
